# A national survey of therapeutic facilities for managing hypoxic ischaemic encephalopathy in tertiary neonatal wards in South African public hospitals

**DOI:** 10.7196/sajch.2025.v19i4.2917

**Published:** 2025-12

**Authors:** N N Dhlomo, M S Pepper, J van Rensburg, S Pillay, A R Horn

**Affiliations:** 1 Division of Neonatal Medicine, Department of Paediatrics and Child Health, Groote Schuur Hospital, Faculty of Health Science, University of Cape Town, South Africa; 2 Institute for Cellular and Molecular Medicine, Department of Medical Immunology, Faculty of Health Sciences, University of Pretoria, South Africa; 3 Extramural Unit for Stem Cell Research and Therapy, South African Medical Research Council, Pretoria, South Africa

**Keywords:** neonate, newborn, hypoxia, ischaemia, brain, hypothermia, induced, Africa South of the Sahara, neonatal encephalopathy, aEEG

## Abstract

**Background.:**

A 2011 South African (SA) survey noted substantial variability in provision of therapeutic hypothermia (TH) for moderate-severe neonatal encephalopathy due to suspected hypoxic-ischaemic encephalopathy (NESHIE).

**Objectives.:**

To describe facilities, including TH, for babies with NESHIE in SA tertiary/specialised public hospitals.

**Methods.:**

A survey was emailed to representatives of all public tertiary/specialised hospitals with tertiary neonatal beds in SA. The survey contained questions regarding bed numbers, equipment, staffing, follow-up and barriers to the implementation of TH.

**Results.:**

Responses were received from all 25 hospitals across the nine provinces. Most hospitals (*n*/*N*=22/25; 88%) provided TH (median (range) per province: 2 (1 – 8)). The hospitals providing TH had more total neonatal beds (*p*=0.011) and more admissions with moderate-severe NESHIE (*p*=0.008) compared with those not providing TH. Most cooling centres (*n*/*N*=20/22; 91%) provided TH in their NICUs; six of these also cooled at lower levels of care. There was access to amplitude integrated electroencephalography (aEEG) and automated cooling in 91% of cooling centres. Manual cooling was used by 50% if automated TH was not available, 82% of which were validated methods. Late arrival was the most frequent barrier to provision of TH (80%).

**Conclusion.:**

All provinces and most tertiary/specialised hospitals with tertiary neonatal beds in SA provided TH in 2023. The use of acceptable monitoring and cooling methods was widespread but insufficient, and some cooling methods in use were not validated. National registers and standardised protocols for patient selection and management are needed.

Therapeutic hypothermia (TH) is the only evidence-based intervention which can decrease both mortality and neurodevelopmental delay following moderate-severe hypoxic-ischaemic encephalopathy (HIE).^[1]^ Neonatal encephalopathy (NE) is diagnosed when a depressed level of consciousness and abnormal tone occurs in term and near-term neonates, with or without additional signs. When NE occurs soon after birth and is associated with the need for resuscitation at birth, as well as signs or investigations suggesting acute peripartum or intrapartum hypoxia (acute or partial/prolonged), it is termed HIE.^[2]^ Since other conditions may co-exist and/or masquerade as HIE, the terminology, ‘intrapartum-related NE’ has been proposed.^[3]^ The terminology, ‘NE due to suspected HIE’ (NESHIE) was subsequently adopted by South African (SA) clinicians as it clearly describes the presumed diagnosis and expected management.^[4–6]^ The incidence of NESHIE is higher in SA than the estimated global average of 1.5 per 1 000 live births.^[7]^ A population-based study in the southern Cape Peninsula of data from 2009,^[3]^ reported an incidence of HIE (defined by criteria from TH trials) of 2.9 /1 000 live births in public facilities, and a hospital-based study of data from 2011 in Gauteng reported an incidence of 8.5/1 000 live births – in both studies the incidence varied with different definitions of intrapartum hypoxia.^[3,8]^

The management of babies with NESHIE includes maintaining metabolic and cardiorespiratory stability, and the use of TH. In 2010, the International Liaison Committee on Resuscitation (ILCOR) recommended that babies born at 36 or more weeks’ gestational age (GA) with evolving moderate to severe HIE should be offered TH, using protocols similar to those used in published clinical trials in facilities with the capabilities for multidisciplinary care and longitudinal follow-up.^[9]^ This recommendation was further refined by the 2015 International Consensus on Cardiopulmonary Resuscitation and Emergency Cardiovascular Care Recommendations, where the committee stated that TH may be considered in resource-limited settings if there are adequate facilities.^[10]^

SA cohort studies have shown the feasibility of low-technology cooling methods,^[11]^ and the methodology for severity classification using robust clinical methods and amplitude-integrated electroencephalography (aEEG),^[12]^ to allow objective selection of babies for cooling,^[13]^ and prognostication by 48 hours of life.^[14]^ A survey of SA paediatricians conducted in 2011, found that only 51% of participants offered TH or transferred babies for TH. However, 98% stated that TH should be the standard of care in tertiary neonatal units.^[15]^ A large observational study in the Western Cape, published in 2016, described outcomes comparable with those seen in high-income countries.^[16]^ However, when the SA genetic-based study of babies with NESHIE commenced in 2019,^[4]^ the use and methodology of TH was anecdotally highly variable throughout SA.

## Objectives

The primary study objective was to describe the facilities for the care of babies with NESHIE in SA tertiary/specialised public hospitals with tertiary neonatal beds.

The secondary objectives were to: (*i*) establish the capacity to manage babies with NESHIE in relation to the availability of equipment, beds and staffing; (*ii*) provide a limited update to a previous survey on the use of TH in SA hospitals; and (*iii*) determine the availability of tertiary/specialised hospitals with tertiary neonatal beds in South Africa.

## Methods

### Design, setting and participants

A cross-sectional, web-based Research Electronic Data Capture (REDCap) survey was conducted from July 2023 to September 2023, across 25 SA tertiary/specialised public hospitals with tertiary neonatal beds, including hospitals from all nine provinces. The classification of hospitals was based on the National Health Act of 2003, amended March 2012;^[17]^ we included specialised hospitals defined as maternity hospitals with tertiary neonatal beds, and tertiary hospitals which provided specialist and subspecialist services, including maternal and neonatal intensive care. The list of hospitals and heads of units (HOUs) were known to the authors from national association meetings and confirmed prior to commencement of the research.

The quantitative survey, which included 22 multiple choice and 17 closed numerical/categorical questions (supplementary file), was emailed to HOUs or designated representatives of the neonatal units. The survey sought information on burden and definition of moderate to severe HIE, the numbers of funded level three (tertiary) neonatal beds, staffing ratios, equipment, the use of TH and availability of follow-up facilities. Data were provided by participating hospitals with permission of the hospital manager and/or Chief Executive Officer (CEO).

The email which invited participation explained the purpose of the research and stated that by participating voluntarily, consent was implied. In addition, the email stated that the research was approved by the University of Pretoria (ref. no. 418/2017, Amendment 12) and the University of Cape Town (UCT) Human Research Ethics Committee (HREC) as a sub-study of the NESHIE study (HREC ref. no. 622/2018), with independent additional approval as a separate study (UCT HREC ref. no. 622/2018). Approval was also obtained from the management of each of the participating hospitals directly. The email further stated that while individual participants and hospitals may be known to the researchers, there would be no individual identifiers in publications. However, the differences between the provinces would be documented. Automated reminders were sent to non-responders until the survey closed at the end of September 2023.

### Data analysis

The data were entered by participating centres, each with a unique code, directly onto a password-protected relational REDCap database and exported onto a Stata spreadsheet which was kept on a password-protected computer. Stata version 17 (Stata Corp., USA) was used for statistical analysis. Data were analysed using basic descriptive statistical methods and presented in tables and charts. Continuous variables were described as median, interquartile range (IQR) and range, owing to the small sample and heterogeneity of participating centres. Categorical variables were described as frequencies. Data were grouped according to provision of TH and compared using Chi-square or Fisher’s exact tests for categorical variables and Wilcoxon rank-sum for continuous variables. Statistical significance was assigned as *p*<0.05.

## Results

Responses were received from clinicians at all 25 hospitals, representing all nine provinces in South Africa (SA). Gauteng had the most tertiary/specialised public service hospitals with tertiary neonatal beds (*n*/*N*=8/25; 32%), followed by KwaZulu-Natal (*n*/*N*=4/25; 16%). The Western Cape and Eastern Cape had 12% (*n*/*N*=3/25) each, Free State and Mpumalanga had 8% (*n*/N=2/25) each, and the provinces with the lowest numbers were Northern Cape, Limpopo and North West, with only one hospital (4%) each. Seventeen responses (68%) were from neonatologists. Most surveys (*n*/*N*=18/25; 72%) were completed by the HOU, the majority of whom were neonatologists (*n*/*N*=14/18; 72%). Twenty-two (88%) of the surveyed hospitals provided TH and at least one hospital per province provided TH (median 2, range 1 – 8). In addition to the TH provided in the surveyed hospitals, which were all tertiary/specialised hospitals, the respondents indicated knowledge of TH provision at other levels of care: level one hospital, 1 province; level two hospital, 3 provinces; and regional hospital, 6 provinces.

### Levels of care and equipment in hospitals providing therapeutic hypothermia

The settings and equipment used in the surveyed hospitals which provided TH are shown in Table 1. Most hospitals (91%) provided TH in NICU; however, six of these hospitals also provided TH at lower levels of care. Two hospitals only provided TH in high care with capacity for nasal continuous positive-airway pressure (nCPAP). Most hospitals (91%) also used automated cooling machines and aEEG; however, the majority indicated more equipment was needed to deliver an optimal TH service. Half of the respondents used manual methods, either as the only method for TH, or as an additional method; 82% used validated manual methods (i.e. servo-control gel-pack cooling method or MiraCradle (Dräger, Germany)).

### Burden of disease and facilities for intensive care and follow-up

The burden of disease and facilities for intensive care and follow-up in centres providing TH compared with centres who do not, are shown in Table 2. One hospital from each category did not provide NESHIE admission data. The hospitals providing TH had more total neonatal beds (*p*=0.011) and estimated significantly more admissions with moderate-severe NESHIE (*p*=0.008) compared with hospitals who did not provide TH. However, both categories had similar capacity for invasive blood pressure (BP) monitoring, ventilation and inhaled nitric oxide (iNO). All hospitals had beds with capacity and equipment for ventilation. There were a total of 252 ventilator beds in cooling centres, and 27 in non-cooling centres. Intubated (invasive) ventilation during the first 24 hours was available in most (92%) hospitals for babies with moderate NESHIE, but only 72% provided intubated ventilation when NESHIE was severe. The proportions of hospitals with ventilation available for babies with moderate and severe NESHIE, and the baby-to-nurse ratio for ventilator beds were not significantly different relative to provision of TH.

Total parenteral nutrition (TPN) was available in all hospitals, and the use of peripherally inserted central catheters (PICC) was frequent and similar in the two groups. There was also similar availability of cerebral ultrasound (CUS) and aEEG machines; however, magnetic resonance imaging (MRI) was routinely performed in more hospitals that did not provide TH (*p*=0.004). Neurodevelopmental follow-up services were available in 96% of hospitals.

### Barriers to provision of optimal therapeutic hypothermia

**Fig. 1.**
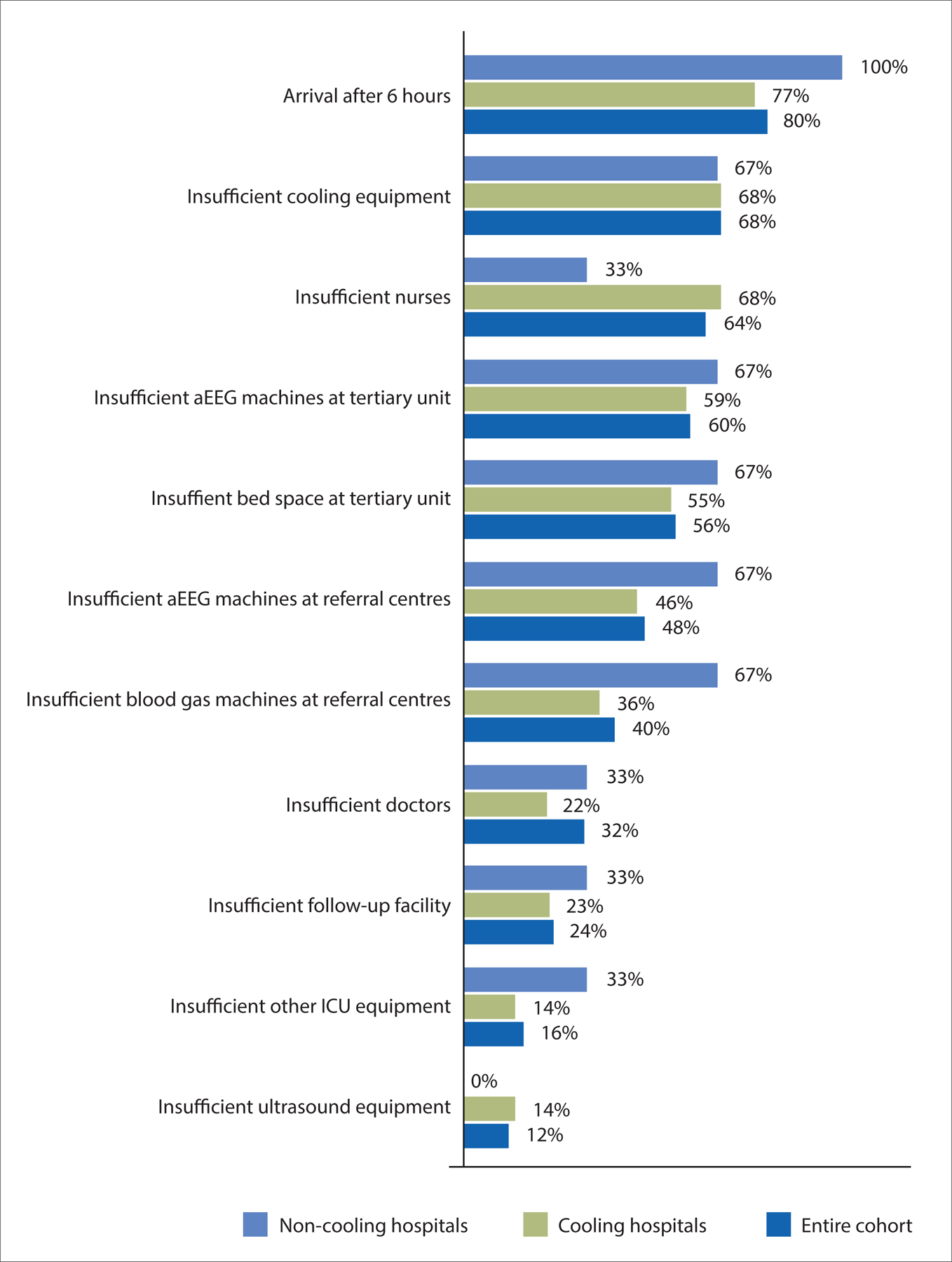
Barriers to provision of optimal therapeutic hypothermia: cooling v. non-cooling centres. (aEEG = amplitude-integrated electroencephalography.)

The perceived barriers to provision of optimal TH in centres providing TH compared with those who do not, are shown in order of frequency in Fig. 1. All hospitals listed at least one barrier. There were no significant differences between hospitals which provide TH and hospitals which do not. Arrival of babies referred for cooling after 6 hours of life was most frequent (80%), followed by insufficient cooling equipment, insufficient nursing staff, lack of bed space, lack of monitoring equipment at treatment and referral centres (centres which refer to tertiary/specialised hospitals), lack of blood gas machines at referral centres, insufficient doctors, follow-up facilities and other NICU equipment.

## Discussion

The present survey of facilities for babies with NESHIE, in SA tertiary/specialised public hospitals with tertiary neonatal beds, had a 100% response rate. Most hospitals provided TH, with at least one hospital from each province providing TH. The majority of hospitals providing TH used acceptable and validated monitoring and cooling equipment, but most noted the need for more equipment and staff to optimally manage babies with NESHIE.

The high response rate, and the high proportion of respondents who were HOUs and/or neonatologists, shows appropriate representation and expertise which validate the findings. The response rate was higher than a 2011 cross-sectional web-based survey of SA paediatricians in both public and private practice regarding NESHIE management, where less than half (37%) of polled clinicians responded.^[15]^ The higher response rate in our survey is probably due to a smaller target group, limited to public institutions where each participant was identified in advance. The lowest number of tertiary/specialised hospitals were predominantly in provinces with more rural populations such as the North West and Northern Cape – these areas are likely to have the greatest challenges in terms of healthcare resources and distances between centres. Several hospitals indicated TH was done in other institutions, at lower levels of care, which were not surveyed by this study – these practices are important to survey separately, since there is no evidence to support cooling without intensive monitoring.

### Therapeutic hypothermia practices and equipment

The widespread adoption of TH (88%) in SA public hospitals represents a substantial increase compared with 56.5% in 2011.^[15]^ The increase may represent previous inclusion of different levels of care compared with the tertiary context of this survey. The increase may also reflect the frequent national workshops in South Africa during the last decade, an increasing number of subspecialty training sites with increased registered neonatologists, the availability of SA protocols for management of NESHIE, ^[18]^ and the 2015 ILCOR recommendations.^[10]^ The use of automated cooling machines is prevalent, similar to protocols in high-income countries (HIC).^[19]^ However, many centres use manual cooling methods, with the majority (82%) being validated manual methods. Validated low-technology cooling methods are acceptable and have been shown to improve outcome in the Infant Cooling Evaluation (ICE) trial,^[20]^ and also in a systematic review and meta-analysis by Rossouw *et al*.,^[21]^ where low-technology methods reduced mortality within intensive care settings in LMICs. Conversely, the use of non-validated methods targeting pharyngeal temperature is unreliable,^[22]^ and fan cooling without a validated device or long-term data is not appropriate – these methods are not recommended. Several centres provide TH in high care where nCPAP is the maximum respiratory support offered – and some centres provided TH in both settings. The data for efficacy in this setting are still accumulating but a recent SA cohort study showed only 17% mortality in cooled babies, which is comparable with other SA data.^[16,23]^ The use of TH at levels lower than high care is not recommended. The demonstration of the pressure to cool outside of the NICU setting, and the need for additional equipment highlights the imbalance between the burden of disease and resources.

### Burden of disease and NICU capacity

The present survey indicated that hospitals offering TH had higher bed numbers and more babies with moderate-severe NESHIE, defined by the Total Body Hypothermia For Neonatal Encephalopathy (TOBY) Trial criteria,^[24]^ or less restrictive criteria.^[3]^ However, considering the similar availability of critical care resources and a lower burden of disease at hospitals who do not cool, TH should be feasible at these centres. The decreased availability of ventilation for babies with severe NESHIE, together with the recent report from Gauteng, showing limited capacity to cool babies with severe NESHIE and poor outcomes of severe NESHIE,^[23]^ highlight the need for a standardised, pragmatic approach to managing NESHIE.

The 23 centres which provided estimates of the burden of moderate-severe NESHIE (by TOBY criteria), indicated a total of 1 098 babies with moderate-severe NESHIE per annum, equivalent to 4 392 ventilator-bed-days assuming 4 days in a ventilator-bed is required. The total number of ventilator-beds, across all 25 assessed centres, was 279 – equivalent to 101 835 ventilator-bed-days per annum. These data suggest that if all babies presenting with moderate-severe NESHIE at the tertiary/specialised hospitals were managed in ventilator-beds, they would require at least 4% of the annual ventilator-bed-days across the 25 centres.

However, these data should be contextualised within the national burden of moderate-severe NESHIE, defined by criteria used in TH RCTs; the incidence was 2.5/1 000 live births in the southern Cape Peninsula in 2009,^[3]^ and varied from 4.7 – 6.1/1 000 live births in Gauteng from data spanning 2011 – 2019.^[8,23]^ Based on these data, the SA national incidence may be conservatively estimated as 5.5 per 1 000 live births, or 4 666 babies per annum, based on 848 337 reported live births in 2023.^[25]^ These estimates suggest there are at least 3 538 babies with NESHIE who are not reaching tertiary/specialised hospitals. Our survey did not extend to regional hospitals and other centres which may be cooling some of these babies, but the resource challenges at tertiary/specialised hospitals are probably more prevalent at regional centres. These findings indicate the need for a broader national review of capacity and management.

### Neurological assessment and follow-up

Most hospitals had access to aEEG, CUS and neurodevelopmental services, indicating appropriate diagnostic evaluation and follow-up. Paradoxically, a greater proportion of hospitals not providing TH conducted routine MRIs compared with hospitals providing TH. Although MRI is the gold standard to assess brain injury in babies with HIE,^[26]^ utilisation is constrained by cost, limited accessibility and minimal impact on subsequent management. The more widely accessible CUS is helpful to assess the brain and exclude pre-existing abnormalities.^[26]^ A similar paradox was noted in a recent global survey, where more LMICs conducted MRI and neurodevelopmental assessment compared with HICs.^[27]^

### Barriers to provision of optimal TH

According to ILCOR, TH should be available if there are adequate infrastructure and resources for appropriate supportive care.^[10]^ The barriers identified in our survey, particularly arrival time after 6 hours and staffing and equipment constraints, demonstrate the geographical, socioeconomic and management challenges in providing or optimising TH. A retrospective review at Charlotte Maxeke Johannesburg Academic Hospital similarly cited arrival after the 6-hour therapeutic window and lack of equipment as reasons for not providing TH,^[28]^ and delay in initiation of TH within the 6-hour window is associated with worse outcomes.^[14]^ The high proportion of out-born babies (70%) in the Hypothermia for Encephalopathy in LMICs (HELIX) study may explain the increased mortality in cooled babies (42% v. 31% in the control group; *p*=0.022) and the lack of benefit in the combined outcome of death or disability.^[29]^ The authors of the HELIX study suggested that TH should not be offered in LMICs. However, this suggestion has been challenged by SA neonatologists and others, citing the high proportion of out-born babies, missing intrapartum data, the inclusion of growth-restricted babies, the high incidence of funisitis, and the increased illness severity compared with SA cohorts.^[30,31]^ Strategies to address the barriers identified in this survey and to minimise the potential pitfalls of the HELIX trial should include increasing and standardising resources, reviewing and standardising patient selection criteria and management protocols, and optimising coordination between referral and tertiary centres.

### Strengths and limitations

The 100% response rate with strong sub-specialist representation is a major strength. Further strengths are the identification of education gaps, as well as demonstration of the mismatch between the burden of disease and resources. The exclusion of non-tertiary/specialised hospitals is a limitation; however, the present study provides important foundational data.

## Conclusion

This survey highlights the implementation of TH in SA public tertiary/specialised hospitals over the last decade, showing that all provinces and most tertiary/specialised hospitals provide TH in 2023. The use of recognised monitoring and cooling methods was widespread but insufficient, and a minority of cooling methods were not validated. The organisational and socioeconomic barriers to implementation of TH indicate the need for national standardised cooling protocols. The variations and challenges provide a foundation for future research, which should focus on identifying babies who will benefit the most from TH, identifying reasons for not offering TH, exploring alternative methods of neuroprotection, and establishing a national register to track management, patient profiles and outcomes.

## Supplementary Material

Supplementary File

## Figures and Tables

**Table 1.** Settings and equipment in cooling centres (*N*=22)

Variable	*n*/*N* (%)*
Cooling done in NICU – proportion of cooling centres	20/22 (91)
Cooling done in high care with capacity for nCPAP – proportion of cooling centres	8/22 (36)
Cooling done in lower levels of care at cooling centres – proportion of cooling centres	1/22 (5)
Number of functional aEEG machines available per cooling centre, median (IQR) (range)^†^	1 (1 – 2) (0 – 8)
Number of additional aEEG machines needed to continuously monitor all babies per cooling centre, median (IQR) (range)^‡^	2 (2 – 3) (0–10)
aEEG available at any referral hospitals (hospitals referring to the cooling centres)	4/22 (18)
Number of fully functional automated cooling machines available, per cooling centre^§^	1 (1 – 3) (0 – 5)
Number of additional automated cooling machines required to cool all babies with HIE, per centre^‡^	2 (1 – 3) (0 – 8)
**Centres that used manual methods of cooling – proportion of cooling centres**	11/22 (50)
• Centres that used validated manual cooling methods:	
Servo-control gel pack cooling method	8/11 (73)
• MiraCradle	1/11 (9)
**Other manual methods used**	
• Ice packs and pharyngeal temperature monitoring	1/11 (9)
• Fan cooling with rectal probe monitoring	1/11 (9)

NICU = neonatal intensive care unit; nCPAP = nasal continuous positive-airway pressure; aEEG = amplitude-integrated electroencephalography; IQR = interquartile range; HIE = hypoxic ischaemic encephalopathy.

* Unless otherwise specified.

† Functional aEEGs – indicates working and available to use if consumables are available at the time of the survey.

‡ Based on the maximum number of infants at any time who met cooling criteria.

§ Functional cooling machines – indicates working and not awaiting repairs or consumables.

**Table 2.** Burden of disease, facilities and follow-up in all surveyed centres

Variable	Entire cohort (*N*=25), median (IQR)*	Hospitals providing TH (*n*=22), median (IQR)*	Hospitals not providing TH (*n*=3), median (IQR)*	*p*-value
**Annual burden of disease** * ^†^	***N*=23**	***N*=21**	***N*=2**	
Moderate-severe HIE defined by TOBY criteria	40 (25 – 52)	40 (30 – 52)	2 (0 – 3)	0.008
Range	0 – 170	6 – 170	0 – 3	
Moderate-severe HIE with less restrictive criteria	50 (30 – 100)	52 (45 – 100)	4 (2 – 6)	0.008
Range	2 – 180	10 – 180	2 – 6	
**NICU ventilation and monitoring capacity** *^†^	**N=25**	**N=22**	**N=3**	
Total neonatal beds in unit	50 (39 – 73)	62 (44 – 75)	24 (23 – 32)	0.011
Range	10 – 185	10 – 185	23 – 32	
Beds with invasive BP monitoring	12 (6 – 23)	12 (6 – 24)	18 (6 – 23)	0.897
Range	0 – 70	0 – 70	6 – 23	
Beds with intubated (invasive) ventilation	10 (6 – 12)	10 (6 – 12)	6 (6 – 15)	0.834
Range	3 – 60	3 – 60	6 – 15	
Beds with capacity for HFOV	5 (2 – 9)	4 (2 – 9)	6 (1 – 15)	0.944
Range	0 – 60	0 – 60	1 – 15	
**Capacity for nCPAP and iNO** * ^†^	***N*=24**	***N*=21**	***N*=3**	
Beds with capacity for nCPAP	15 (10 – 23)	12 (10 – 24)	15 (15 – 18)	0.785
Range	6 – 78	6 – 78	15 – 18	
Beds with capacity for iNO	0 (0 – 2)	0 (0 – 1)	0 (0 – 15)	0.831
Range	0 – 15	0 – 10	0 – 15	
**Funded level 3 beds** * ^†^	***N*=20**	***N*=18**	***N*=2**	
Median (IQR)	24 (8 – 40)	28 (7 – 40)	24 (23 – 24)	0.897
Range	5 – 110	5 – 110	23 – 24	
**Baby: nurse ratio for ventilator beds** * ^†^	***N*=21**	***N*=18**	***N*=3**	
Median (IQR)	2 (1.5 – 2.5)	2 (1.5 – 3)	1.5 (1 – 2.5)	0.415
Range	0.3 – 3.3	0.3 – 3.3	1 – 2.5	
**NICU support** ^†^	***N*=25**	***N*=22**	***N*=3**	
Intubated ventilation available for severe HIE 1st 24 hours *n/N* (%)^‡^	18/25 (72)	16/22 (73)	2/3 (67)	1.000
Intubated ventilation available for moderate HIE 1st 24 hours, *n/N* (%)	23/25 (92)	21/22 (95)	2/3 (67)	1.000
TPN available, *n/N* (%)	25/25 (100)	22/22 (100)	3/3 (100)	1.000
PICCs used, *n/N* (%)	19/25 (76)	16/22 (73)	3/3 (100)	0.554
**Neurological assessment and follow-up** * ^†^	***N*=25**	***N*=22**	***N*=3**	
Neurodevelopmental follow-up service, *n/N* (%)	24/25 (96)	21/22 (96)	3/3 (100)	1.000
Functional aEEG machines	1 (1 – 2)	2 (1 – 2)	1 (1 – 1)	0.324
Range	0 – 8	0 – 8	1 – 1	
Access to CUS, *n/N* (%)	23/25 (92)	20/22 (91)	3/3 (100)	1.000
Routine MRI, *n/N* (%)	4/25 (16)	2/22 (9)	2/3 (67)	0.004

IQR = interquartile range; HIE = hypoxic ischaemic encephalopathy; TOBY = Total Body Hypothermia For Neonatal Encephalopathy Trial; NICU = neonatal intensive care unit; HFOV = high-frequency oscillation ventilation; nCPAP = nasal continuous positive-airway pressure; iNO = inhaled nitric oxide; TPN = total parenteral nutrition; PICC = peripherally inserted central catheter; aEEG = amplitude integrated electroencephalography; CUS = cranial ultrasound; MRI = magnetic resonance imaging.

* Unless otherwise specified. (IQR and range specified owing to small numbers.)

† *N* in the sub-heading represents number of participants who answered.

‡ Refers to the proportion of hospitals offering ventilation during the first 24 hours of life, since some hospitals will offer a period of limited intervention.
