# Supplementary material for: A national survey of therapeutic facilities for managing hypoxic ischaemic encephalopathy in tertiary neonatal wards in South African public hospitals
Source: SAJCH. Author manuscript; Available in PMC 2026 Jul 9. (PMC13343329; doi:10.7196/sajch.2025.v19i4.2917)
Supplement: Supplementary File [file NIHMS2189514-supplement-Supplementary_File.pdf]

Appendix 1: Questions for NESHIE survey

Therapeutic facilities for managing Hypoxic Ischaemic Encephalopathy in tertiary neonatal wards in South African public hospitals: A national survey

Institutional details
1. What is the name of your hospital?
2. What is your position (select one - head of neonatal unit/neonatologist/paediatrician)
3. Has the CEO or COO or equivalent given permission for you complete this survey AND if you are not the head of the neonatal unit, has the head of the unit given permission for you to complete this survey? (answer must be "yes" to continue)
4. Would you like to receive the published (de-identified) survey data for your personal records?

Patients requiring cooling
5. Do you provide therapeutic hypothermia as a treatment for NESHIE
6. According to TOBY criteria, how many infants at a gestational age of 36 weeks or more with moderate-severe NESHIE do you estimate will / would be cooled in your unit per year? *(Note: A summarised description of the TOBY criteria will be presented prior to asking this question.)*
7. How many infants at a gestational age of 36 weeks or more with moderate-severe NESHIE do you estimate will / would be cooled in your unit per year if the Apgar score criteria were changed to a 10-min Apgar score of less than 7 and the BD was changed to 10 or more (as in the Shankaran cooling study)?

Equipment & Facilities: aEEG
8. How many functional aEEG machines do you have in your NICU?
9. Will your staff be able to apply and ensure the integrity of aEEG sensors?
10. How many types of aEEG machines do you use in your NICU?
11. Please select all types of aEEG machines used in your NICU
12. Please indicate the main type of aEEG in use in your NICU
13. Please indicate the next most commonly used type of aEEG machine used in your NICU
14. How many more aEEG machines will you need to ensure continuous aEEG monitoring on all cooled infants in your NICU?

Equipment & Facilities: Cooling machines
15. How many FUNCTIONAL automated cooling machines do you have in your NICU?
16. Please select all types of automated cooling machines used in your NICU
17. Please choose the main type of automated cooling machine used in your NICU
18. Please choose the next most commonly used type of automated cooling machine in your cooling unit
19. How many more automated cooling machines would you need to ensure cooling of all infants meeting cooling criteria in your cooling unit?
20. Will your staff be able to manage automated cooling machines?

Equipment & Facilities: CUS & MRI options
21. Do you have access to a cranial ultrasound machine to allow you to do at least one within the first 24 hours and one ultrasound before discharge?
22. Do you routinely obtain MRI on all infants with suspected HIE admitted to your unit?

Equipment & Facilities: Staff & Treatment capacity

23. How many level 3 beds in your unit?
24. What percentage of level 3 beds in your NICU have multiparameter monitoring with capacity for invasive BP monitoring?
25. What percentage of level 3 beds in your NICU have capacity for invasive ventilation?
26. What percentage of level 3 beds in your NICU have capacity for HFOV?
27. What percentage of level 3 beds in your NICU have capacity for iNO?
28. What percentage of level 3 beds in your NICU have capacity for nCPAP?
29. Is TPN available in your unit?
30. If TPN is available is it routinely administered within the first 24 hours of life?
31. Please estimate the proportion of babies with NESHIE who receive TPN per year.
32. Do you make use of PICC lines in your unit?
33. Does your NICU have, or refer to, a neurodevelopmental follow-up service?
34. Does your NICU refer complex neonatal cases to a higher level unit?
35. Does your NICU refer surgical neonatal cases to a surgical unit?
36. What is the average nurse:patient ratio for the level 3 beds (if no-one is on a break)?
37. What is the average doctor:patient ratio for the level 3 beds (if no-one is on a break), excluding specialists and subspecialists?
38. Is TH provided at other hospitals within your GSA? If so, please list them
39. Is aEEG monitoring available at hospitals which refer to you? If so, please list them.
